# Safe sleep practices in a New Zealand community and development of a Sudden Unexpected Death in Infancy (SUDI) risk assessment instrument

**DOI:** 10.1186/1471-2431-14-263

**Published:** 2014-10-13

**Authors:** Barbara C Galland, Andrew Gray, Rachel M Sayers, Anne-Louise M Heath, Julie Lawrence, Rachael Taylor, Barry J Taylor

**Affiliations:** Department of Women’s & Children’s Health, University of Otago, Dunedin, New Zealand; Department of Preventive and Social Medicine, University of Otago, Dunedin, New Zealand; Department of Human Nutrition, University of Otago, Dunedin, New Zealand; Department of Medicine, University of Otago, Dunedin, New Zealand

**Keywords:** Bed sharing, Breastfeeding, Environmental risk factors, Maternal depression, Prone sleeping, SIDS, Parental smoking

## Abstract

**Background:**

Interventions to prevent sudden unexpected death in infancy (SUDI) have generally been population wide interventions instituted after case–control studies identified specific childcare practices associated with sudden death. While successful overall, in New Zealand (NZ), the rates are still relatively high by international comparison. This study aims to describe childcare practices related to SUDI prevention messages in a New Zealand community, and to develop and explore the utility of a risk assessment instrument based on international guidelines and evidence.

**Methods:**

Prospective longitudinal study of 209 infants recruited antenatally. Participant characteristics and infant care data were collected by questionnaire at: baseline (third trimester), and monthly from infant age 3 weeks through 23 weeks. Published meta-analyses data were used to estimate individual risk ratios for 6 important SUDI risk factors which, when combined, yielded a “SUDI risk score”.

**Results:**

Most infants were at low risk for SUDI with 72% at the lowest or slightly elevated risk (combined risk ratio ≤1.5). There was a high prevalence of the safe practices: supine sleeping (86-89% over 3–19 weeks), mother not smoking (90-92% over 3–19 weeks), and not bed sharing at a young age (87% at 3 weeks). Five independent predictors of a high SUDI risk score were: higher parity (*P* =0.028), younger age (*P* =0.030), not working or caring for other children antenatally (*P* =0.031), higher depression scores antenatally (*P* =0.036), and lower education (*P* =0.042).

**Conclusions:**

Groups within the community identified as priorities for education about safe sleep practices beyond standard care are mothers who are young, have high parity, low educational levels, and have symptoms of depression antenatally. These findings emphasize the importance of addressing maternal depression as a modifiable risk factor in pregnancy.

## Background

Sudden unexpected death in infancy (SUDI) is a broad term used for all sudden unexpected infant deaths ranging from those that remain unexplained after a full investigation (unexplained SUDI) to those where a full explanation of the death is found during subsequent investigations (explained SUDI). Sudden infant death syndrome (SIDS) and “cot death” have previously been used to describe the “unexplained SUDI” group where SIDS is defined as “the sudden and unexpected death of an infant under 1 year of age, with onset of the lethal episode apparently occurring during sleep, that remains unexplained after a thorough investigation including performance of a complete autopsy, and review of the circumstances of death and the clinical history” [1]. In developed countries, unexplained SUDI represents the highest proportion of all post-neonatal deaths [2].

In the late 1980s and early 1990s, education programs and campaigns, commonly referred to as the “Back to Sleep” campaigns, were started after several risk factors for SIDS were discovered, most importantly prone (front) sleeping, smoking, bed sharing (particularly in the presence of maternal smoking), and not breastfeeding. These programs were followed by a dramatic decline in unexplained SUDI [3, 4] supporting the idea that such risk factors might be causally related [5]. In New Zealand (NZ), rates of unexplained SUDI have continued to decline, but have always been relatively high compared to other countries [6]. The unexplained SUDI rates per 1000 live births amongst Māori (the indigenous people of NZ) have been 5 times that of non-Māori [7]. However a recent decline in unexplained SUDI and among Māori in NZ has been reported [8], the reasons for which remain to be determined.

The first aim of this study was to determine the extent to which infant care practices for prevention of SUDI are being followed in a NZ community, as much of what is known about trends in practices linked to SUDI reduction in NZ has been derived from surveys conducted in the 1990s and early 2000s. The second aim was to develop a SUDI risk assessment instrument that could be used to identify maternal, infant and household variables predictive of SUDI risk. Such a risk score could be used in identifying cases where additional support for families is needed and might indicate useful points for interventions to target, as well as facilitating comparisons between communities and within communities over time.

## Methods

Information was collected about the infant care practices of 209 Dunedin, NZ, parents of infants born between June 2009 and February 2011. Participants were families comprising the control group (n =209) of a 4-arm randomized controlled trial (RCT): the Prevention of Overweight in Infancy (POI) study (total n =802). Parents were recruited antenatally from the single maternity hospital servicing Dunedin city. Infants were excluded if they lived outside the study area, were born before full term (36.5 weeks), or if a congenital abnormality or a physical or intellectual disability likely to affect feeding, physical activity or growth was identified. In total, 1458 of 2946 women were eligible to participate in the RCT. After declines (n =511) and post-birth exclusions (n =45), 802 enrolled in the main trial (58% response rate). However only data from the control group (n =209) were included here because the intervention arms received education and support on infant sleep and/or breastfeeding. Thus the control group received standard care, whereas the 3 intervention groups received 1) breastfeeding, activity and complementary feeding education and support, 2) infant sleep education, or 3) both interventions. The RCT study details including group allocation methods have been published [9]. The New Zealand Lower South Regional Ethics Committee approved the study (Project Key: LRS/08/12/063) and all participants gave written informed consent.

### Information for parents on safe sleep

All NZ families receive information about infant safe sleep practices via the standard care offered free and delivered by registered Well Child providers, during home or clinic visits typically scheduled at ages 6 weeks, and 3 and 5 months. A range of issues, including safe sleep are covered at these sessions and Well Child providers are required to provide proof to the NZ Ministry of Health that such issues have been discussed [10]. Midwives, handing over care to Well Child providers at 6 weeks, also follow Ministry guidelines in regard to informing parents about infant safe sleep practices. In addition, written materials on helping protect babies against SUDI are given to parents at antenatal and postnatal visits.

### Data collection

At baseline (third trimester) and monthly at infant ages 3 weeks through 23 weeks, parents completed questionnaires collecting data on the variables to be used to calculate the SUDI risk score: sleep position, place of sleep, smoking, breastfeeding, and pacifier use. Questions were also asked about bedding (under and over baby). The questionnaires were administered in person at baseline, 3 and 19 weeks (full questionnaires), and by telephone at 7, 11, 15 and 23 weeks (using a subset of questions to minimize participant burden). Additional data collected at baseline were: demographic information, pre-pregnancy body mass index (BMI), maternal depression (using the 10-item Edinburgh Postnatal Depression Scale (EPDS) [11] validated for use in the prenatal period [12]), mother’s report of parenting stress (using the attachment and adaptability sub-scales from the Parenting Stress Index (PSI) [13]), and maternal alcohol consumption via a brief screening 3-question test for heavy drinking and active alcohol abuse or dependence, the Alcohol Use Disorders Identification Test (AUDIT-C) [14]. The NZ Deprivation Index (NZDep2006) [15] was used as an index of neighborhood deprivation based on the participant’s address at baseline. The index range is 1 to 10, with 1 representing areas of least deprivation, and 10, areas of highest deprivation. Infant birth characteristics were collected from hospital records following birth.

### Development of the SUDI risk assessment instrument

Five key “best practice” variables were identified (sleeping supine, not smoking during pregnancy, not bed sharing, breastfeeding and using a pacifier) based on NZ and international guidelines and research on SUDI prevention. Estimated risk ratios (with odds ratios used to approximate these given the low prevalence of SUDI) were extracted from the literature for not following each “best practice”: sleep supine (back) [16], not smoking during pregnancy [17], not bed sharing (calculating separate risks for those who smoked and those who did not) [18], breastfeeding (any) [19], and using a pacifier [20]. The last of these is not currently part of NZ guidelines around SUDI prevention. The SUDI risk scores were calculated for each family in the study, using data collected at the age when each “best practice” was most relevant. We then created a total risk score for participants by multiplying these risk ratios together if they were not following one or more of the best practice recommendations. For example, an infant sleeping prone was given an OR of 6.91 for this practice [16]. If the same infant had a mother who smoked during pregnancy, but did not bed share, an OR of 1.98 [18] was also given. The risk ratio (relative risk) for the infant was then calculated at 13.7, i.e. the product of the odds ratios for the two practices. An estimated risk ratio of 1 is the reference value (all best practice). Adjusted odds ratios were used where possible but were not available for prone/side sleep position or for breastfeeding.

### Statistical analysis

Appropriate summary statistics for sleep practices of interest and the SUDI risk scores are presented. The numbers of participants contributing to each variable of interest are described within the Tables. Cases with missing data for a particular variable were omitted for the unadjusted and any adjusted analyses involving that variable. Ethnicity was prioritized in order of Māori, Pacific, Asian, Other, and finally European. This order of prioritization follows national recording standards used when a participant responds with more than one ethnicity. Infant ethnicity was based on further prioritizing both maternal and partner ethnicity using the same ordering. SUDI risk scores were calculated as described earlier. Linear regression was used to explore predictors of SUDI risk scores. Unadjusted models were developed for the following variables relating to the mother: age, prioritized ethnicity (in order of Māori, Other, European), education, self-reported pre-pregnancy BMI, parity, EPDS scores [11], PSI scores [13], and AUDIT-C scores [14]; relating to the household: NZDep2006 [15] and family income; and relating to the infant: gestational age and sex. Variables with unadjusted *P* <0.25 were included in a final adjusted model. Fractional polynomials were used to investigate, and where present, model non-linear associations. Standard model diagnostics were investigated including normality and homoscedasticity of residuals. A log-transformation for the risk score was used to reduce skew and heteroscedasticity in model residuals and so effects are shown as ratios of geometric means, alongside 95% CIs, and *P*-values. All analyses were conducted using Stata 13.1 (StataCorp. 2013. *Stata Statistical Software: Release 13*. College Station, TX: StataCorp LP) and two-sided *P* <0.05 was considered statistically significant.

## Results

Table 1 summarizes the maternal, infant, and household characteristics. The majority of infants were classified as European (77.5%) or Māori (11.5%). Approximately 70% of mothers had received a post-secondary education, 93% lived with the infant’s father/partner and 45% of families lived in neighborhoods within the mid-range of the deprivation index (NZDep deciles 4–7). Family income was not reported by 8%, but of those remaining, 44% received more than the average household income for the region at the time of the study.

**Table 1:** **Maternal, family, and infant characteristics**

	Total (n)	n (%)	Mean (SD)
Maternal age at birth (years)	209	-	31.5 (5.0)
Maternal prioritized ethnicity	209	-	-
European	-	177 (84.7)	-
Māori	-	15 (7.2)	-
Other^c^	-	17 (8.2)	-
Maternal BMI (pre-pregnancy) categories	207	-	-
Underweight	-	9 (4.4)	-
Healthy	-	113 (54.6)	-
Overweight	-	57 (27.5)	-
Obese	-	28 (13.5)	-
Maternal education	206	-	-
Year 11 or lower	-	14 (6.8)	-
Year 12 or 13	-	41 (19.9)	-
Post-secondary	-	29 (14.1)	-
Degree or higher	-	122 (59.2)	-
Maternal employment	209	-	-
Working (full-time, part-time, or casual)	-	120 (57.4)	-
Maternity leave (paid or unpaid)	-	33 (15.8)	-
Student (and possibly working)	-	7 (3.4)	-
Not working (includes carers)	-	49 (23.4)	-
Parity	209	-	2 (1)^a^
Maternal EPDS	208	-	6.8 (4)
Maternal stress	207	-	12.7 (3.4)
Maternal AUDIT-C	162	-	0 (1)
Living arrangements	208	-	-
With child’s father/partner	-	193 (92.8)	-
With family	-	10 (4.8)	-
Other	-	5 (2.4)	-
NZDep2006	206	-	-
1-3 (low deprivation)	-	74 (35.9)	-
4-7	-	93 (45.2)	-
8-10 (high deprivation)	-	39 (18.9)	-
Family income (NZD^d^)	209	-	-
≤$30,000	-	20 (9.6)	-
-$70,000	-	80 (38.3)	-
Over $70,000	-	92 (44.0)	-
Not provided	-	17 (8.1)	-
Gestation (weeks)	209	-	40.0 (1.3)
Birth weight (g)	207	-	3522 (484)
Infant sex (M:F)	209	0.88^b^	-
Infant prioritised ethnicity	209	-	-
European	-	162 (77.5)	-
Māori	-	24 (11.5)	-
Other^c^	-	23 (11.0)	-

^a^median (IQR).

^b^ratio.

^c^Other includes Pacific, Asian, Middle Eastern, Latin American, African.

^d^NZD, New Zealand dollars. $70,000 represents average annual household income in region.

Unless otherwise stated, maternal and family data were collected at baseline (third trimester).

### Infant care practices, breastfeeding and parental smoking

**Figure 1:**
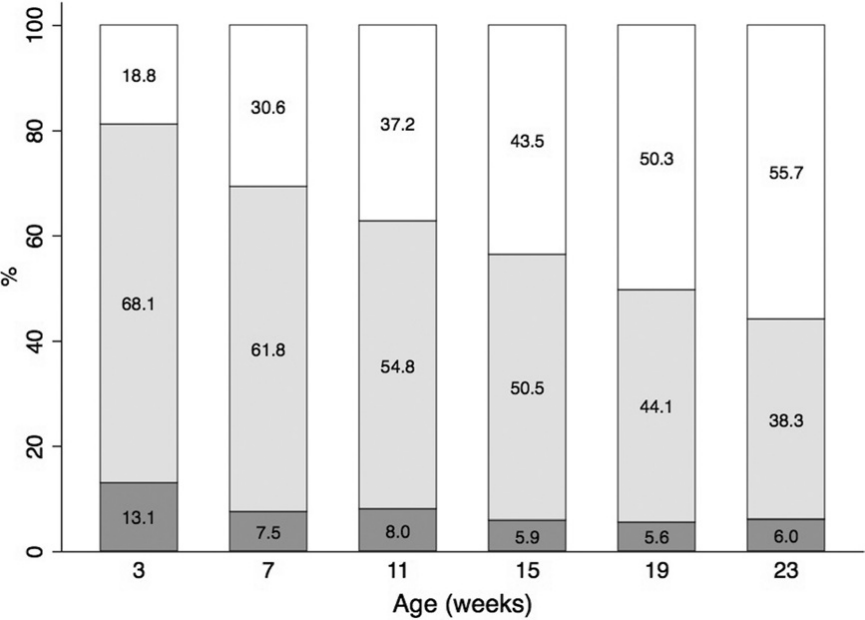
**Change in infant nighttime sleep location from 3 to 23 weeks.** Percentage of infants bed sharing (dark shading), room sharing (light shading), and sleeping in their own room (white).

The safest sleep position, supine (back), was highly prevalent with 86% and 90% of infants sleeping in this position at 3 and 19 weeks respectively. Only 2.0% and 2.1% chose to sleep their baby prone (front) at 3 and 19 weeks respectively. Figure 1 shows the changes in sleep location from infant age 3 weeks to 19 weeks. Thirteen percent of participants reported bed sharing at 3 weeks of age, reducing by almost half to 7.5% at 7 weeks and 5.6% at 19 weeks of age. Room sharing in a cot or bassinette at 3 weeks was the most common practice (68%), transitioning to infant sleeping in a separate room over time.

At approximately 2 months of age, 89% of infants were receiving at least some breast milk. The majority (71%) of those being breastfed at that age were breastfed exclusively. No specific education is given about pacifier use in NZ but we include it here because it is a safe sleep message adopted by other countries. Within our cohort, pacifier use was relatively uncommon with 10% of infants using a pacifier daily at 3 weeks rising to 17%, 18%, 19%, 19% and 19% at 7, 11, 15, 19 and 23 weeks respectively. Eight percent and 10% of mothers were smokers (current or daily) in the third trimester of pregnancy, and at infant age of 19 weeks, respectively.

Of additional interest is the finding that 18% of partners were smokers in the third trimester of pregnancy, and a similar figure at an infant age of 19 weeks. The prevalence of smoking in the car was low (mothers 2.5% and 1.1% during pregnancy and at infant age 19 weeks respectively, and partners, 2.3% and 6.6% respectively) as was smoking inside the home. Another practice that is discouraged, but that was not included in the SUDI risk score, was using a sheepskin as a soft surface under bedding, which was used by 10% of families. The use of plastic wrapping under the bedding was uncommon at 4.2%.

### Best practice and the SUDI risk ratio assessment

Best practice variables related to SUDI prevention, and the data source and odds ratios (95% CIs) for individual risks are given in Table 2. In our sample, best practices predominated for all risk factors excluding pacifier use. For example, supine sleeping was practiced by 90%, any breastfeeding by 89%, and not bed sharing in combination with no maternal smoking by 81%. As only 1 mother smoked during pregnancy and bed shared with her infant, the prevalence of this risk factor in our sample was less than 0.5%. Consequently, risky practices were low. The only (internationally) recommended practice that few parents followed was regular use of a pacifier (19%).

**Table 2:** **Data source for SUDI Risk score and the number of infants in this study following best practice**

			Current study				
Best practice	Risk factor	Data source	Risk score odds ratio (95% C.I.)	Age (weeks)	n	Following best practice, n (%)	
						Yes	No
Supine sleep position	Prone or side sleep position	Gilbert et al., 2005 [16]	6.91 (4.63-10.32)	19	195	176 (90.3)	19 (9.7)
Any breastfeeding	No breastfeeding	Hauck et al., 2011 [19]	2.63 (1.85-3.7)^a^	8.7	199	178 (89.4)	21 (10.6)
Usual pacifier use	No pacifier use	Hauck et al., 2005 [20]	1.41 (1.12-1.69)^b^	19	194	36 (18.6)	158 (81.4)
No bed sharing & No maternal smoking in pregnancy				3	191	154 (80.6)	37 (19.4)
	No bed sharing & Maternal smoking in pregnancy	Carpenter et al., 2004 [17]	1.98^b*^	-	-	-	12 (6.3)
	Bed sharing & No maternal smoking in pregnancy	Vennemann et al., 2012 [18]	1.66 (0.91-3.01)^b^	-	-	-	24 (12.6)
	Bed sharing & Maternal smoking in pregnancy	Vennemann et al., 2012 [18]	6.27 (3.94-9.99)^b^	-	-	-	1 (0.5)

^a^unadjusted odds ratios.

^b^adjusted odds ratios.

^*^Mean odds ratio from those not bed sharing and smoking >10 cigarettes per day and those not bed sharing and smoking <10 cigarettes per day.

The SUDI risk score could range from a possible 1.0 (avoiding all risk factors) to 160.6 (with all risk factors present). The arithmetic mean score in this study was 3.1 with a median of 1.4 (values ranged from 1.0-61.0). The frequency distribution was as follows: 20 infants (11%) had minimum risk with an OR =1.0; 106 infants (60%) had an OR of >1 to ≤1.5; 28 infants (16%) had an OR of >1.5 to ≤3; 13 infants (7.4%) had an OR of >3 to ≤10; 7 infants (4.0%) had an OR of >10 to ≤20; 2 infants (1.1%) had an OR of >20.

### Predictors of high SUDI risk scores

Fourteen maternal, infant and household variables were explored as potential predictors of SUDI risk scores (Table 3). The unadjusted models yielded 9 predictors for further analysis (*P* <0.25) with statistically significantly greater risk suggested for low maternal age, low maternal education, maternal non-working status, high EPDS score, low family income, high parity and statistically non-significant results for ethnicity (maternal), maternal stress, and infant sex. The final adjusted model found 5 statistically significant predictors, i.e. higher risk scores were associated with mothers who had a higher number of previous births (*P* =0.028), were younger (*P* =0.030), were unemployed or not caring for other children at baseline (*P* =0.031), had higher EPDS depression scores at baseline (*P* =0.036), and were less educated (*P* =0.042). Māori ethnicity was not a statistically significant independent predictor for a high SUDI Risk Ratio Score (*P* =0.659) after being almost statistically significant (*P* =0.053) in its unadjusted model.

**Table 3:** **Predictors of SUDI risk scores**

Predictor		Unadjusted			Adjusted ^a^ (n = 170)		
	n	Ratio of geometric means	95% CI	***P***-value	Ratio of geometric means	95% CI	***P***-value
Maternal age at birth (years)	176	-	-	-	-	-	-
Fractional polynomial transformed predictors	-	-	-	< 0.001	-	-	-
20	-	1.00	-	-	-	-	-
25	-	0.23	0.12–0.44	-	-	-	-
30	-	0.16	0.07–0.34	-	-	-	-
35	-	0.16	0.08–0.33	-	-	-	-
40	-	0.17	0.09–0.34	-	-	-	-
Linear predictor	-	-	-	-	0.97	0.95–1.00	0.030
Maternal prioritized ethnicity	176	-	-	0.053	-	-	0.649
European	-	1.00	-	-	1.00	-	-
Māori	-	1.79	1.12–2.88	-	1.21	0.75–1.95	-
Other	-	0.98	0.67–1.43	-	0.92	0.64–1.32	-
Maternal BMI pre-pregnancy (per unit)	174	1.01	0.99–1.03	0.297	-	-	-
Maternal education	173	-	-	< 0.001	-	-	0.042
Year 11 or lower	-	1.00	-	-	1.00	-	-
Year 12 or 13	-	0.50	0.31–0.80	-	0.61	0.37–1.00	-
Post-secondary	-	0.39	0.23–0.64	-	0.52	0.31–0.87	-
Degree or higher	-	0.33	0.21–0.51	-	0.52	0.33–0.83	-
Maternal employment	176	-	-	0.001	-	-	0.031
Not working	-	1.00	-	-	1.00	-	-
Full-time, part-time, casual	-	0.64	0.52–0.79	-	0.79	0.64–0.98	-
Parity (per birth)	176	1.15	1.05–1.27	0.004	1.13	1.01–1.26	0.028
EPDS (per point)	175	1.06	1.03–1.09	< 0.001	1.03	1.00–1.06	0.036
Stress (per point)	175	1.02	0.99–1.06	0.177	1.01	0.98–1.05	0.353
AUDIT-C (per point)	140	0.97	0.84–1.11	0.657	-	-	-
Living arrangements	176	-	-	0.697	-	-	-
Without partner or family	-	1.00	-	-	-	-	-
With partner	-	1.35	0.57–3.19	-	-	-	-
With family	-	1.55	0.56–4.28	-	-	-	-
NZDep2006 (per decile)	173	1.02	0.97–1.06	0.489	-	-	-
Family income (NZD^b^)	176	-	-	< 0.001	-	-	0.062
≤$30,000	-	1.00	-	-	1.00	-	-
-$70,000	-	0.93	0.62–1.39	-	0.94	0.63–1.41	-
Over $70,000	-	0.62	0.41–0.92	-	0.77	0.51–1.17	-
Not provided	-	1.39	0.81–2.37	-	1.38	0.80–2.38	-
Gestation (per week)	176	0.98	0.90–1.07	0.708	-	-	-
Infant sex	176	-	-	0.246	-	-	0.283
Girl	-	1.00	-	-	1.00	-	-
Boy	-	0.88	0.70–1.10	-	0.90	0.73–1.10	-

^a^Adjusted for all other variables in the model.

^b^NZD, New Zealand dollars. $70,000 represents average annual household income in region.

Unless otherwise stated, maternal and family data were collected at baseline (third trimester).

## Discussion

The present study demonstrates that the main safe sleep messages for SUDI prevention are highly practiced within this NZ community sample. Risk scores calculated for our population confirmed that the majority (72%) were at either the lowest or slightly elevated risk (SUDI Risk Ratio Score ≤1.5) for SUDI through safe sleep and feeding practices. Importantly, this score identified 5 predictors of SUDI risk related to the mother: young maternal age, low education, not employed in the third trimester of pregnancy, high parity, and high depression scores antenatally. Most of these factors would be expected, but are difficult to modify. However, the increased SUDI risk score amongst mothers with high depression scores on the EPDS antenatally is of particular interest, given the strong association between antenatal and postnatal depression [21], the adverse consequences of depression for quality maternal-infant interactions (reviewed in [22]), and the reported association of postnatal depression with SUDI [23]. Although there is a high degree of health surveillance antenatally in NZ, screening for maternal depression is not routine.

The SUDI risk assessment instrument is, as far as we are aware, unique within the SUDI literature in that it incorporates a range of risk factors without assuming that each has the same impact. It provides a single, generalizable, easily used score for SUDI risk that does not require clinical measurements and can be easily updated as new evidence becomes available through meta-analyses or large studies. Potential applications include its use as a tool for cross-study comparisons (e.g., across different cultures), and for identifying temporal changes (e.g., assessing the impact of public health campaigns).

One similar tool exists, the SIDS risk index score of Conroy and Marks [24] derived from five high risk SUDI sleep practice variables identified from the Confidential Enquiry into Stillbirths and Deaths in Infancy study [25] in a sample of 66 disadvantaged families, the majority of whom were from an ethnic minority group. Bed sharing was not included, and all risk factors were treated as additive, unlike the current study, which has treated the risk factors as multiplicative as the risk estimates were obtained from odds ratios. Two main findings were similar: not being first-born, and higher psychological vulnerability of the mother, were independent predictors of a high SIDS risk index score [24]. Their measure of psychological vulnerability included the EPDS score at 2 months post-partum. In both studies, the EPDS score was treated as a continuum, rather than as a presence or absence of depression defined using clinical cutoffs. Thus, it is the symptoms of depression, rather than clinical depression per se, that the current study and that of Conroy and Marks [24] emphasize as being an important predictor.

Maternal Māori ethnicity was almost statistically significant as a predictor of a high SUDI risk in the unadjusted model (*P* =0.053), but the association was greatly attenuated after adjusting for other variables (adjusted OR of 1.21 compared to unadjusted OR of 1.79) and was no longer a tendency in the adjusted model. Neither low family income nor household deprivation were independent predictors of SUDI risk.

The prevalence of prone sleeping in our study was low at 1.3% and 2.8% of 3 week and 19 week old infants respectively. Nationwide figures for the prevalence of prone sleeping in 3 month old infants before the “Back to Sleep” campaign were 33% [26], dropping to 3.0% afterwards [27]. The prevalence of side sleeping nationally was 73% after the Back to Sleep campaigns [27]. Side sleeping was later recognized as an additional risk factor for SIDS and education to discourage the practice resulted in a fall in the prevalence with local reports of a prevalence of 21% in 2003 [28]. The present study suggests a further reduction with 12% and 7.6% of infants sleeping on their side at 3 weeks and 19 weeks of age respectively. With the reduction in both side sleeping and prone sleeping, the prevalence of supine sleeping here was 90%, appreciably higher than the 62% reported in 2003 [29]. Major reasons cited for non-supine sleeping positions are infant preference, infant comfort, and parental fear of choking [30]. However, it is clear that the safe sleep position messages prevail within our community.

The prevalence of mothers smoking during pregnancy in this study (8%) is slightly lower than the national average of 11%, although the prevalence is significantly higher nationally amongst Māori women (34%) and in women with lower markers of socioeconomic status [31]. However these groups are not strongly represented in this sample. Meta-analyses published in 1997 [32] and in 2013 [33] concluded that maternal smoking doubles the risk of SIDS. The risks attributed to passive smoking remain unclear, however one study has reported that the risk from postnatal exposure increases with the number of smokers in the household, or with the daily hours the infant is subjected to an environment with cigarette smoke [34]. Encouragingly, in our study, figures for smoking in the car and smoking inside the home were very low.

The advice to not bed share, particularly if the mother smoked in pregnancy or currently, was also being adhered to in this study with only one case of bed sharing in a mother who smoked. However, bed sharing was twice as common at 3 weeks of age as at 7 weeks and older. The reason for this higher rate in younger infants is unknown but may be due to parental choice, maternal sleep needs, or infant feeding practices. A large meta-analysis identified that bed sharing in the absence of maternal smoking was still a risk for SUDI (at 2 weeks, odds ratio =2.4) but was only significant during the first 8 weeks of life [17]. Furthermore, the risk of bed sharing infants dying in a maternity hospital bed within the first few days of birth has received attention [35]. Room sharing with the baby sleeping in its own cot or bassinette for the first 6 months of life is the recommended practice. Anecdotally, some parents say this is difficult to comply with and Figure 1 illustrates the shift in this practice as the infant gets older, with 68% room sharing at 3 weeks declining to 38% at 23 weeks.

Pacifier use at sleep time is associated with a lower SUDI risk through a yet to be determined mechanism [36]. Despite some countries recommending pacifier use for SUDI prevention [37], NZ does not include pacifier use as part of its safe sleep messages. We found pacifier use more than doubled from 3 weeks of age [9.6%] to 15 weeks, [19%], similar to the 19% of 3 month old infants in this region using pacifiers in 2001/2 [29]. This suggests no significant effect of international recommendations on pacifier use in this community, and no broader social effect even though pacifier use is much more common in other countries [32% to 71%] [38].

The suggestion that breastfeeding may be protective against SUDI has been controversial, with many, although not all, studies reporting an association [39]. However, a recent meta-analysis suggests that breastfeeding is protective, and that the effect is even stronger when breastfeeding is exclusive [19]. Eighty-nine percent of infants in the current study were breastfed at 2 months of age, compared to 79% of NZ infants aged approximately 6–9 weeks [40]. Although exclusive breastfeeding is likely to provide greater protection against SUDI [19], we used “any breastfeeding” at 2 months as the marker for protective breastfeeding behaviour. This was because the only estimate available in the literature of the relative risk of SUDI amongst infants who are breastfed was for “any breastfeeding” at 2 months [19].

The strengths of this study include the comprehensive longitudinal dataset, and the availability of earlier data with which to compare and describe changes in child-care practices over time. When exploring independent predictors for SUDI risk scores, this study was able to take into account a wide range of maternal infant and family characteristics. Limitations include, first, the respondents were from areas of low SUDI risk and predominantly Caucasian, thus we don’t know how well the score would translate to a study of high-risk infants. Second, the SUDI risk assessment instrument included some unadjusted odds ratios. As data with adjusted odds-ratios for all variables become available from future meta-analyses or high-quality studies, a better estimate of the risk will be possible. Third, the calculation of the total SUDI risk score assumes multiplicative and not additive risks. While this seems appropriate given that the score is based on odds ratios, which are multiplicative in nature, we don’t know if this is the case for all factors considered here and it is possible that correlations between risk factors not adjusted for in the analyses found in the literature may lead to very high risk estimates for some families. Again, further calibration of the instrument will be possible when better estimates become available. Finally, missing data limited the calculation of risk scores to 176 of the 209 participants recruited into this arm of the study (84.2%). Missing alcohol data reduced the number of observations available for that unadjusted model by a further 36 and biases in answering this question may have affected the association reported here. However, as AUDIT-C was not included in the adjusted model, this did not substantially affect the sample size available for that model which was reduced by only 6 (3.4%) due to missing covariates. It seems plausible that missing risk score data would be largely missing at random after conditioning on the covariates included in the adjusted model. However, the presence of missing data increased the widths of the presented confidence intervals and some non-statistically significant predictors (ethnicity and income) have confidence intervals which do not rule out important associations.

## Conclusions

This study has identified groups within the community as priorities for education about safe sleep practices beyond standard care: young mothers, mothers with high parity, mothers with low educational levels, and mothers with symptoms of depression antenatally. The antenatal period is an opportune time to screen for symptoms of depression, providing the chance for early intervention and treatment before birth. We reinforce earlier data suggesting maternal depression is a marker for SUDI risk [23] and within the context of our findings, related to infant care practices. Further research is required to determine whether the findings also apply to infants at high risk of SUDI, particularly in ethnic minority populations, with different cultural infant care practices.

## Abbreviations

AUDIT-C: Alcohol use disorders identification test
EPDS: Edinburgh post-natal depression scale
NZDep2006: New Zealand deprivation index 2006
PSI: Parental stress index
SIDS: Sudden infant death syndrome
SUDI: Sudden unexpected death in infancy.
